# The regulatory ZFAS1/miR-150/ST6GAL1 crosstalk modulates sialylation of EGFR via PI3K/Akt pathway in T-cell acute lymphoblastic leukemia

**DOI:** 10.1186/s13046-019-1208-x

**Published:** 2019-05-16

**Authors:** Qianqian Liu, Hongye Ma, Xiuhua Sun, Bing Liu, Yang Xiao, Shimeng Pan, Huimin Zhou, Weijie Dong, Li Jia

**Affiliations:** 1 College of Laboratory Medicine, Dalian Medical University, Dalian 116044, 9 Lushunnan Road Xiduan, Dalian, 116044 Liaoning Province China; 2 Department of Clinical Laboratory, Beijing Hospital of Traditional Chinese Medicine Affiliated to Capital University of Medicine Sciences, Beijing, 100010 China; 3 Department of Medicine Oncology, Second Affiliated Hospital of Dalian Medical University, Dalian, 116027 Liaoning Province China; 4 Department of Microbiology, Dalian Medical University, Dalian, 116044 Liaoning Province China; 5 Department of Biochemistry, Dalian Medical University, Dalian, 116044 Liaoning Province China

**Keywords:** T-ALL, ST6GAL1, miR-150, ZFAS1, EGFR/PI3K/Akt pathway

## Abstract

**Background:**

Noncoding RNAs, including microRNAs (miRNAs) and long non-coding RNAs (lncRNAs) are becoming key parts in the development of multidrug resistance (MDR) in T-cell acute lymphoblastic leukemia (T-ALL). Abnormal expression in sialyated *N*-glycans have been observed in MDR leukemia. However, the role of sialylation regulated MDR remains poorly understood. The aim of this work is to analyze the alternation of *N*-glycans in T-ALL MDR.

**Methods:**

Here, mass spectrometry (MS) is analyzed to screen the *N*-glycan profiles from ALL cell line CR and adriamycin (ADR)-resistant CR (CR/A) cells. The expression of sialyltransferase (ST) genes in T-ALL cell lines and bone marrow mononuclear cells (BMMCs) of T-ALL patients were analyzed using qRT-PCR. Functionally, T-ALL cell proliferation and MDR are detected through CCK8 assay, colony formation assay, western blot and flow cytometry. RIP assay and Dual-luciferase reporter gene assay confirm the binding association between ZFAS1 and miR-150. Xenograft nude mice models are used to determine the role of ST6GAL1 in vivo.

**Results:**

Elevated expression of α2, 6-sialyltransferase 1 (ST6GAL1) has been detected. The altered level of ST6GAL1 was corresponding to the drug-resistant phenotype of T-ALL cell lines both in vitro and in vivo. ZFAS1/miR-150/ST6GAL1 axis was existed in T-ALL cell lines. MiR-150 was downregulated and inversely correlated to ST6GAL1 expression. ZFAS1 was a direct target of miR-150 and positively modulated ST6GAL1 level by binding miR-150. ZFAS1/miR-150/ST6GAL1 axis functioned to regulate ADR-resistant cell growth and apoptosis. Besides, EGFR was demonstrated to be a substrate of ST6GAL1, and the sialylated EGFR had an impact on the PI3K/Akt pathway.

**Conclusion:**

Results suggested that ZFAS1/miR-150/ST6GAL1 axis involves in the progression of T-ALL/MDR further mediates sialylated EGFR via PI3K/Akt pathway. This work might have an application against T-ALL MDR.

**Electronic supplementary material:**

The online version of this article (10.1186/s13046-019-1208-x) contains supplementary material, which is available to authorized users.

## Background

T-cell acute lymphoblastic leukemia (T-ALL) is abnormal proliferation of lymphocytes in hematopoietic system, characterized by high white blood cell counts and infiltration of immature T lymphocytes into the bone marrow and other tissues. Despite recent development in T-ALL treatment, approximately 10% children and 60% adult with T-ALL develop a terrible prognosis [1]. Among all the T-ALL patients who relapse after long-term chemotherapy, drug resistance to a wide range of chemotherapeutic agent constitutes a cause, leading to multidrug resistance (MDR) [2]. Therefore exploring the mechanism of drug resistance in T-ALL is necessary and of great urgency.

It has been known that tumor cells display an altered profile of cell surface glycans. Sialic acids are carboxylated monosaccharides on glycosylated proteins and lipids because of post translational modification. Increasing evidence indicates that abnormal sialylation is closely related to malignant tumor phenotypes, including proliferation, invasion, metastasis and drug-resistance [3–6]. Sialytransferases (STs) are a family of anabolic enzymes, consisting of 20 members that are subjected into three families. These glycosyltransferases could convert sialic acid from cystidine- 5-monophospho-N-acetylneuraminic acid to glycoproteins or glycolipids. Alpha-2, 6-sialytransferases mediate the transfer of SA with an alpha 2, 6-linkage to it with terminal GAL (ST6GALI, II). Evidence has demonstrated that altered sialylation involves in a variety of biological processes, including cell-cell communication, cell-matrix interaction, adhesion and protein targeting ST6GAL1 is the main sialyltransferase responsible for α2, 6-linked sialic acid formation on *N*-glycans, which is highly associated with poor outcomes [7]. ST6GAL1 could induce anti-apoptosis and drug resistance to help tumor cells escape from the influence of chemotherapeutic drugs [8, 9]. ST6GAL1 was also found increased in ALL [10], but the underlying mechanism remains unclear. Moreover, there is limited information regarding the molecular details of how α2, 6-sialylated proteins, catalyzed by ST6GAL1, mediate T-ALL function.

A number of non-coding RNAs have been identified, including miRNAs and lncRNAs, which participate in the regulatory network of hematopoietic malignancies [11]. MiRNAs are 19–25 nucleotides to regulate the translation by targeting specific messenger RNA (mRNAs). Many studies have shown that miRNAs are involved in leukemia [12]. Downregulation of miR-150 has been observed in CML and acute AML [13]. LncRNAs are a class of RNA molecules longer than 200 nucleotides without protein-coding potential. Aberrant expression of lncRNAs could play important regulatory roles in cell proliferation, differentiation, and apoptosis [14–16]. Recent studies reveal that ZFAS1 is highly expressed in hepatocellular carcinoma and colorectal cancer, promotes metastasis and tumorgenicity by involving in cell cycle [17, 18]. ZFAS1 also promotes metastasis by binding miR-150 to abrogate tumor-suppressive function [19]. However, the special function of ZFAS1 modulates MDR in T-ALL by sponging miR-150 is not deeply studied.

Epidermal growth factor receptor (EGFR), which is a transmembrane receptor tyrosine kinase (RTK), activates multiple pathways. EGFR could be activated by ST6GAL1, turning out to be a substrate of ST6GAL1 in ovarian cancer [20]. Inhibition of EGFR pathway by various inhibitors enhanced anticancer drugs induced cell death in T-ALL cells [21]. Phosphatidylinositol 3-kinases (PI3K)/Akt are a group of signaling transduction enzymes involved in the production of intracellular second messenger lipid signals. In many tumors, continued activation of PI3K/Akt pathway has been implicated as a central role in the proliferation, survival and drug resistance [22]. Furthermore, the PI3K pathway is activated in 92% of T-ALL cell lines and 81% of primary T-ALL samples, respectively [23]. So PI3K and the downstream protein-serine/throenine kinase which are frequently mutated in T-ALL cells are important therapeutic targets in T-ALL.

In this study, the expression pattern of *N*-glycan in T-ALL cell lines was analyzed, and the increased expression of ST6GAL1 in T-ALL cell lines was positively correlated MDR. ZFAS1 was regarded as a competing endogenous RNA to regulate ST6GAL1 by sponging miR-150. EGFR was α2, 6 sialylated glycoprotein, catalyzed by ST6GAL1. ZFAS1/miR-150/ST6GAL1 axis regulated proliferation and chemoresistance through sialylated EGFR/PI3K/Akt in T-ALL.

## Methods

### Cell culture and clinical samples

Human T-ALL cell lines, CCRF-CEM (CR) and Jurkat cells (JK) were purchased from the KeyGEN Company (Nanjing, China). They were cultured in RPMI-1640 medium (Gibco, Grand Island, NY) containing 10% heat inactivated fetal bovine serum (Gibco, Grand Island, NY) at 37 °C in a humidified atmosphere of 5% CO2. The drug-resistant cell lines were cultured with adriamycin (ADR, Sigma) with concentration gradually increased until 1 μg/ml to become resistant. The resistant cells were named as CCRF-CEM/ADR (CR/A) and Jurkat/ADR (JK/A). CR/A cell and JK/A cell were incubated with ADR (1 μg/ml) to remain drug resistance.

A total of 46 patients with diagnosis of T-ALL from July 2014 to January 2018 were included from the First Affiliated Hospital of Dalian Medical University. The patients’ information was in Additional file 1: Table S1. The diagnosis of T-ALL was based on cytomorphology, cytochemistry, molecular genetics, multipara meter flow cytometry and immunology. Peripheral blood mononuclear cells (PBMCs) of T-ALL were isolated by Ficoll-Hypaque density gradient separation (Sigma-Aldrich) and then were cultured in plastic dishes to remove adherent cells at 37 °C for 24 h. According to the expressional level of P-gp with fluorescence intensity ≥20%, PBMCs were divided into two groups, T-ALL without MDR and T-ALL/MDR. All experiments were approved by the Institutional Ethics Committee of the First Affiliated Hospital of Dalian Medical University.

### Mass spectrometric analysis

For MALDI-TOF-MS analysis, membrane proteins were extracted using CelLytic MEM Protein Extraction kit (Sigma, St. Louis, MO, USA). The Micro BCA Protein Assay kit (PIERCE, Rockford) was utilized to detect the membrane protein concentration. To release *N*-glycans, three 100 μg aliquots of lyophilized cell membrane protein were digested and dissolved at 37 °C for 18 h. The digest product was bearing a water bath (85 °C, 5 min). After cooling, the *N*-linked glycans were released from peptides with PNGase F enzyme treatment. The released *N*-glycans were purified using an Oasis HLB cartridge (60 mg/3 ml; Waters) then lyophilized. Permethylation was performed by using the solid NAOH technique.

Permethylated *N*-glycans were analyzed with MALDI-TOF mass spectrometer (Bruker Corp., Billeriaca, MA, USA). For MS analysis, the dried permethylated sample was resuspended and added solidated DHB. The experiments were finished with a 4800 Proteomics Analyzer (Applied Biosystems). All MS spectra were obtained from Na^+^ adductions.

### Real-time PCR analysis

Extraction of total RNA from mononuclear cells used Trizol reagent (Invitrogen, USA). First strand cDNA synthesis was performed using a PrimeScript™ RT reagent Kit (TaKaRa). The cDNA was intensified using SYBR Premix Ex Taq™ II (TaKaRa). MiR-150 was normalized to U6, ZFAS1 and ST mRNA data were normalized to GAPDH. The fold change was calculated with 2^-ΔΔCT^ method. All of the reactions were performed triplicate.

### Cell transfection

PCR production of ST6GAL1 and ZFAS1 were cloned into the expression vector pcDNA3.1 (Invitrogen, USA). MiR-150 mimic, negative control oligonucleotides (miR-NC), miR-150 inhibitor, negative control oligonucleotides (NC inhibitor), small interfering RNA of ZFAS1 (siZFAS1), scramble siRNA of ZFAS1 (siSCR) were purchased from RiboBio (Guangzhou, China). The cells were seeded into 6-well plates. The transfection was carried out using Lipofectamine 3000 (Invitrogen, Carlsbad, CA, USA). The transfection efficiency was evaluated by fluorescence microscopy by calculating the percentage of fluorescein-labeled cells.

### Western blot analysis

Cells proteins were collected and equal amounts of total protein were separated using 10% SDS-PAGE and transferred to a polyvinylidenedifluoride membrane (Millipore, Bedford, MA, USA) by electroblotting. Membrane was blocked for 2 h in Tris-buffered saline Tween-20 containing 5% skimmed milk and then probed with different primary antibodies overnight at 4 °C. After 2 h incubation with anti-rabbit IgG, the protein level was detected using ECL Western blot kit (Thermo Fisher Scientific, USA), and analyzed by LabWorks (TM ver4.6, UVP, BioImaging Systems, NY, USA).

### Lectin pull-down assay

Cell lysate was incubated with SNA-agarose. Samples were incubated at 4 °C overnight. Next, α2–6 sialylated proteins were precipitated by centrifugation and washed 3 times with ice cold PBS. Precipitates were resolved by SDS-PAGE and immunoblotted for EGFR as described above.

### Flow cytometry (FCM) analysis

Cells were placed in sterile conical tubes in aliquots of cells each and stained with FITC-SNA lectin antibody at a final concentration of 10 μg/ml for 40 min at 4 °C in the dark. Labeled cells were centrifuged at 1000 r/min, and resuspended in 0.2 ml PBS. They were analyzed with FACS Calibur flow cytometer. Fluorescence intensity was measured by Cell Quest software.

The apoptosis assay was measured using Annexin-V-FITC apoptosis detection kit (BD, Franklin Lakes, NJ, USA). Rates of apoptotic cell were measured by FACS Calibur flow cytometer (Becton-Dickinson, CA, USA), detecting the fluorescence of at least 10,000 cells each sample. Each experiment was run in triplicate.

### Methylcellulose colony formation assay

The test was performed to measure the capacity of cell proliferation. After transfection, 1 × 10^3^ cells were mixed completely with RPM-1640 medium containing 0.9% methylcellulose solution (MethoCult GF M3534), and 10% FBS. They were seeded in six-well plates. After 7–10 days the colony numbers were counted using an inverted microscope (Olympus, Tokyo, Japan). Containing more than 50 cells of the colonies were counted. All experiments were repeated at least three times.

### Cell viability assay

Chemoresistance to ADR was determined by Cell counting Kit-8 (CCK-8; KeyGEN, Nanjing, China). Briefly, cells (1 × 10^4^/100 μl) were plated in 96-well plate with different concentration of ADR. After incubating 48 h in a humidified incubator at 37 °C, 11 μl CCK8 was added into the plate for 4 h. The spectrometric absorbance was measured at 450 nm by microplate reader (168–1000 Model 680, Bio-Rad).

Cell proliferation assay was conducted by CCK-8. Similarly, cells were plated in the same way, and the absorbance was then measured to evaluate the proliferate ability. All analyses were performed in triplicate.

### Dual luciferase assay

Cells were seeded and cultured overnight. Cells were co-transfected with pcDNA3.1 ZFAS1-wt, pcDNA3.1 ZFAS1-mut, pcDNA3.1 ST6GAL1-wt or pcDNA3.1 ST6GAL1-mut was transfected into cells together with miR-150 mimic or the control, respectively. Lipofectamine 3000 (InvitrogenCo, Carlsbad, CA, USA) was used. The luciferase activity was measured after 48 h of transfection using the dual-luciferase reporter gene assay system (Promega, Madison, WI, USA).

### Immunofluorescence staining

Cells were cultured for 12 h in six-well plates (5 × 10^5^ cells per well), then collected and washed three times with cold phosphate-buffered saline (PBS). Cells were fixed with 4% paraformaldehyde for 20 min, treated with 0.2% Triton X-100 for 3 min before incubating with 5% BSA for 1 h. Then, cells were incubated with primary antibody at 4 °C in the dark overnight. The cells were washed with PBS next day and treated with secondary antibody. After incubation with 1 h, cells were then stained with 4′, 6-diamidino2-phenylindole dihydrochloride (DAPI, Sigma-Aldrich, St Louis, MO, USA) for 5 min in the dark. Finally, cells were washed three times with PBS and examined and pictured by fluorescence microscopy (Olympus, Tokyo, Japan).

### Xenografted tumor model

The nude mice (4 weeks old) were purchased from the Model Animal Research Institute of Nanjing University. The mice were randomly assigned to groups (*n* = 6/group). 1 × 10^7^cells were injected into the flank of the nude mice. The tumor volume was examined per week. 28 days later, the tumors were isolated and calculated according to the formula: (length × width^2^)/2.

### Immunohistochemistry staining

Xenograft tumors were collected and immersed in 4% buffered formaldehyde then performed on paraffin-embedded sections. The slides were treated with drying, deparaffining and rehydrating. The slides were immersed with 3% hydrogen peroxide for 10 min and incubated with antibodies overnight at 4 °C. The secondary streptavidin-horseradishperoxidase-conjugated antibody staining (Santa Cruz Biotech, Santa Cruz, CA) was incubated at room temperature for 1 h. The slides were then counterstained with hematoxylin for 30s and cover slipped. Images were taken from a light microscope. The results of the experiments were analyzed by software Image-Pro plus 6.0.

### RNA immunoprecipitation (RIP) assay

RIP assay was performed using the Magna RIP™ RNA Binding Protein Immunoprecipitation Kit (Millipore, Bedford, MA, USA). Cells were collected and lysed in complete RIPA buffer containing a protease inhibitor cocktail and RNase inhibitor. Next, the cell extracts were incubated with RIP buffer containing magnetic bead conjugated with human anti-Ago2 antibody (Millipore) or mouse immunoglobulin G (IgG) control. The samples were incubated with proteinase K to digest proteins. When the RNA was obtained, qRT-PCR was detected to further analyze the presence of binding targets.

### Statistical analysis

The experimental data were analyzed by SPSS software. All data were presented as means ± standard deviation (SD). The one-way analysis of variance (ANOVA) was used to determine the significant difference of multiple groups and Student’s t-test used to evaluate the significant difference of two groups. The survival curves were calculated by Kaplan-Meier method, and the difference was assessed by a log-rank test. The association between miRNA and mRNA expression was identified using Spearman’s correlation analysis. Statistical significance was accepted when *P* value < 0.05.

## Results

### MALDI-TOF MS analysis for N-glycan profiling in T-ALL cell lines

**Fig. 1:**
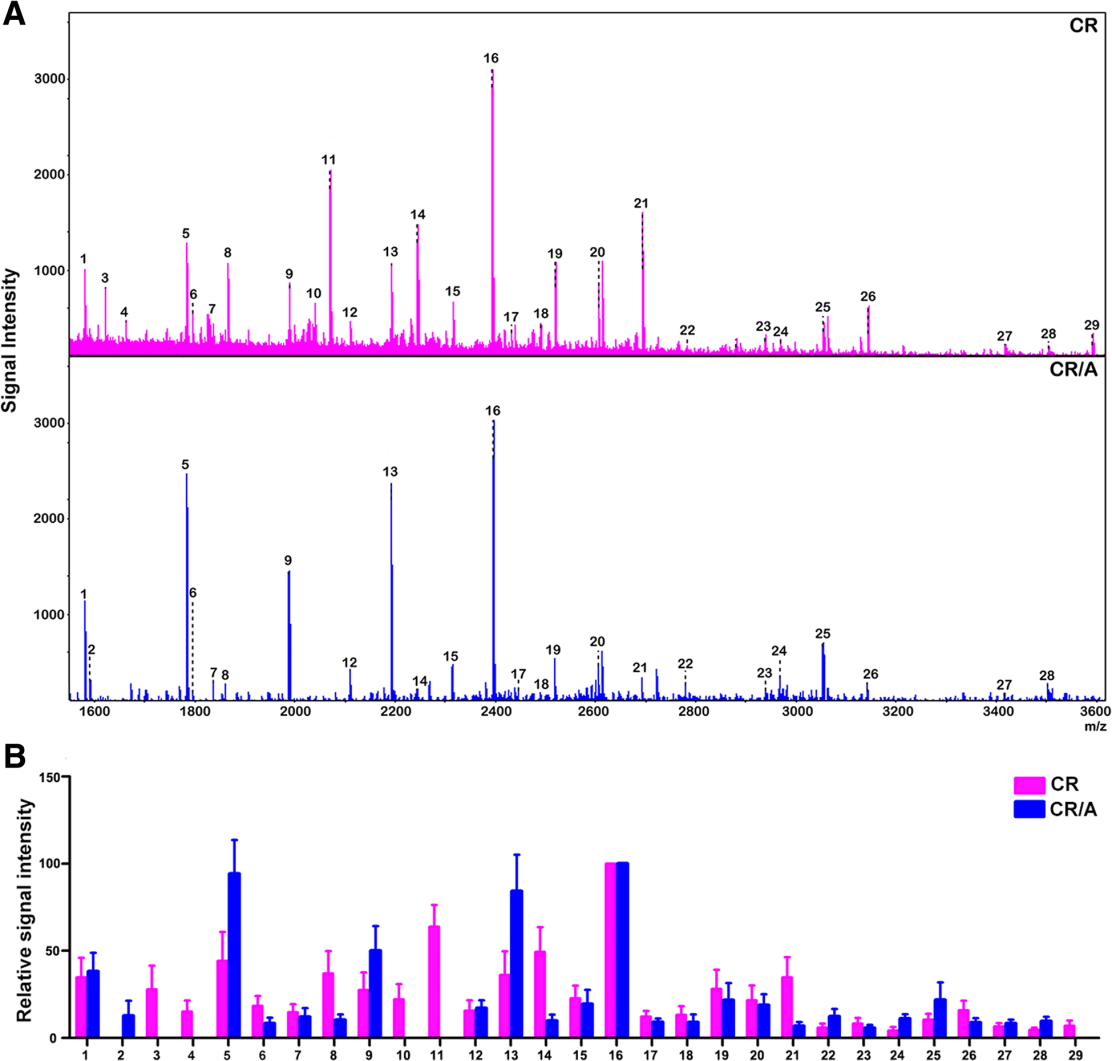
Different *N*-glycan composition in T-ALL cell lines. **a** MALDI-TOF MS spectra of permethylated *N*-glycans released from CR and CR/A cell lines, respectively. **b** Histograms of relative intensities of the differential *N*-glycan signals from both cell lines were observed

Total *N*-glycans from CR and CR/A cells were analyzed and identified using MALDI-TOF MS (Fig. 1a). As summarized in Table 1, 29 kinds of *N*-linked glycans were detected in both cell lines. The *N*-glycans of each fraction in MS spectrum was detected in the m/z range 1000–4000. Histograms of relative expression in *N*-glycan composition produced from three MALDI-TOF MS replicates were shown in Fig. 1b. When the full portraits of *N*-glycan diversity of both cell lines were represented quantitatively, it seems likely that high-mannose *N*-glycans were major components (peaks 1, 5, 9, 13, 16). Six *N*-glycans were identified in both cell lines, all of which were sialylated glycoforms (peaks 17, 20, 22, 24, 25, 28). Furthermore, CR/A cell also exhibited upregulated high-level expression of sialylated glycoforms compared with CR cells (peaks 22, 24, 25, 28, > 2-folds). Thus, MALDI-TOF MS analysis revealed that elevated high sialylated *N*-glycans were presented in ADR resistant cell lines.

**Table 1:** *N*-glcans analyzed in CR and CR/A cell lines by MALDI-TOF MS

Glycan number	Observed *m/z*		Chemical composition
	CR	CR/A	
1	1579.831	1579.752	Man_2_ + Man_3_HexNAc_2_
2		1590.773	HexNAcFuc + Man_3_HexNAc_2_
3	1620.865		HexHexNAc + Man_3_HexNAc_2_
4	1661.908		HexNAc_2_ + Man_3_HexNAc_2_
5	1783.937	1783.842	Man_3_ + Man_3_HexNAc_2_
6	1794.961	1794.850	HexHexNAcFuc + Man_3_HexNAc_2_
7	1835.999	1835.886	HexNAc_2_Fuc + Man_3_HexNAc_2_
8	1886.003	1866.381	HexHexNAc_2_ + Man_3_GlcNAc_2_
9	1988.038	1987.941	Man_4_ + Man_3_HexNAc_2_
10	2040.093		HexHexNAc_2_Fuc + Man_3_HexNAc_2_
11	2070.100		Hex_2_HexNAc_2_ + Man_3_HexNAc_2_
12	2111.132	2110.993	HexHexNAc_3_ + Man_3_GlcNAc_2_
13	2192.148	2192.040	Man_5_ + Man_3_HexNAc_2_
14	2244.200	2244.095	Hex_2_HexNAc_2_Fuc + Man_3_HexNAc_2_
15	2315.250	2315.085	Hex_2_HexNAc_3_ + Man_3_GlcNAc_2_
16	2396.258	2396.139	Man_6_ + Man_3_HexNAc_2_
17	2431.306	2431.827	Hex_2_HexNAc_2_NeuAc + Man_3_HexNAc_2_
18	2489.375	2489.195	Hex_2_HexNAc_3_Fuc + Man_3_GlcNAc_2_
19	2519.345	2519.195	Hex_3_HexNAc_3_ + Man_3_HexNAc_2_
20	2605.397	2605.249	Hex_2_HexNAc_2_FucNeuAc + Man_3_HexNAc_2_
21	2693.444	2693.108	Hex_3_HexNAc_3_Fuc + Man_3_HexNAc_2_
22	2780.595	2779.343	Hex_3_HexNAc_3_NeuAc + Man_3_HexNAc_2_
23	2938.576	2938.816	Hex_3_HexNAc_4_Fuc + Man_3_HexNAc_2_
24	2968.644	2968.458	Hex_2_HexNAc_2_FucNeuAc_2_ + Man_3_HexNAc_2_
25	3054.632	3054.514	Hex_3_HexNAc_3_FucNeuAc + Man_3_HexNAc_2_
26	3142.706	3142.594	Hex_4_HexNAc_4_Fuc + Man_3_HexNAc_2_
27	3417.836	3417.736	Hex_5_HexNAc_5_ + Man_3_GlcNAc_2_
28	3503.995	3503.762	Hex_4_HexNAc_4_FucNeuAc + Man_3_HexNAc_2_
29	3592.045		Hex_5_HexNAc_5_Fuc + Man_3_HexNAc_2_

Abbreviations: *Man* mannose, *Hex* hexose, *HexNAc N*-acetylhexosamine, *Fuc* fucose, *GlcNAc N*-acetylglucosamine, *NeuAc N*-acetylneuraminic acid. The *N*-glycans were observed as [M + Na] +

### ST6GAL1 is upregulated in T-ALL cell lines and T-ALL patients

**Fig. 2:**
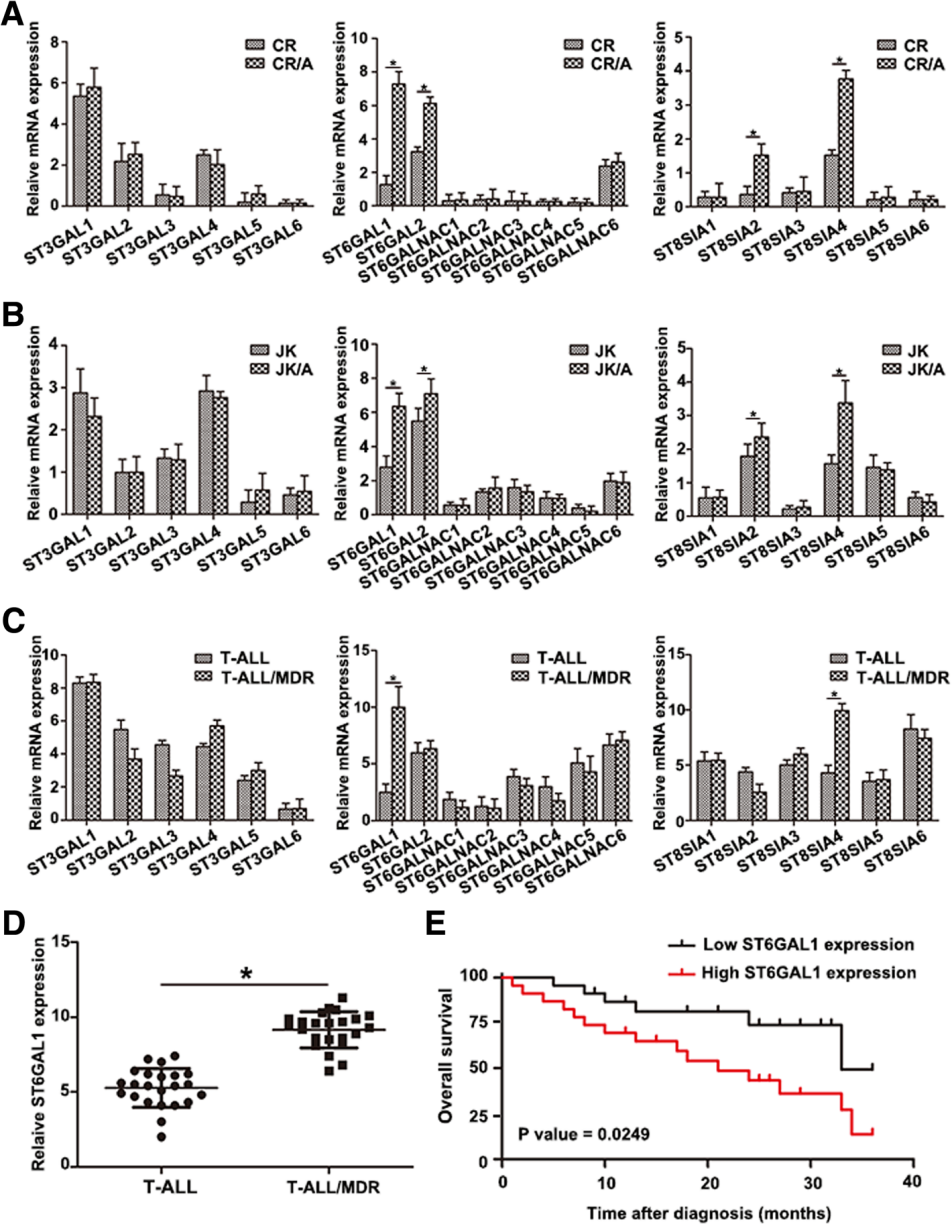
Differential expression of ST6GAL1 in T-ALL cell lines and patients. **a-b** The differential expression of ST gene family was analyzed in T-ALL cell lines by qRT-PCR. **c** The level of STs was tested in T-ALL patients. **d** ST6GAL1 expression was measured in T-ALL and T-ALL/MDR groups (T-ALL: *n* = 23, T-ALL/MDR: n = 23). **e** Kaplan-Meier overall survival curves (OS) was supervised based on the level of ST6GAL1. Data were means ± SD of triplicate determinants (*P < 0.05)

The extent of salic acid type *N*-glycans depended on sialyltransferase. The results showed that the expression of ST gene family was differed between T-ALL cell lines by qRT-PCR. Markable increase of ST6GAL1 was found in ADR-resistant cell lines in Fig. 2a, b. To detect the potential clinical relevance of the observed relationship between ST family and the MDR in T-ALL patients, the expression of ST gene family of PBMC in T-ALL patients was measured (Fig. 2c, *P < 0.05). T-ALL/MDR patients showed higher expression of ST6GAL1 (Fig. 2d). The Kaplan-Meier method was used to analyze the association between ST6GAL1 and overall survival (OS) in T-ALL patients. The results proved that patients with high ST6GAL1 expression had poorer prognosis (Fig. 2e, *P < 0.05). These results showed that ST6GAL1 could be regarded as a potential clinical biomarker to monitor the progression of T-ALL resistance.

### ST6GAL1 influences the chemosensitivity and proliferation of T-ALL cells in vitro and in vivo

**Fig. 3:**
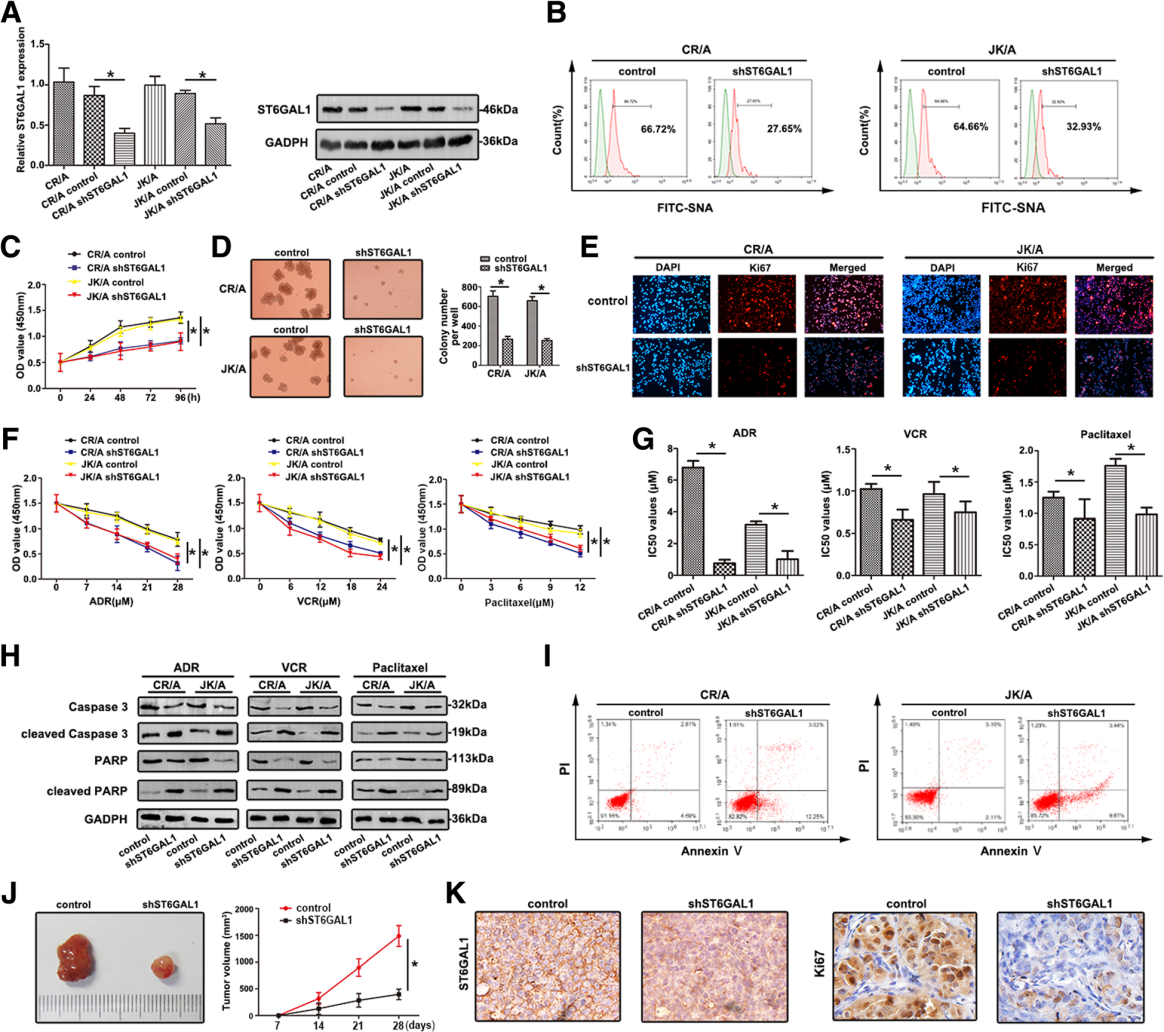
Downregulation of ST6GAL1 attenuates proliferation and chemoresistance in T-ALL cell lines (**a**) The expression of ST6GAL1 was detected by qRT-PCR and western blot. **b** FCM was used to show the sialylation levels on the cell surface of transfected cell lines by FITC-SNA. **c-e** The proliferative ability was performed using CCK-8 assay, colony forming unit assay, immnuofluoresence analysis with Ki67. **f** The chemoresistance to ADR, VCR and Paclitaxel was detected by CCK-8 assays. **g** The IC_50_ values were calculated. **h** The key apoptosis related molecules were determined by western blot. **i** FCM showed the apoptosis of transfected T-ALL cell lines in response to ADR. **j** The tumor tissues of nude mice were presented and calculated. **k** Tumor tissues were sectioned and stained with ST6GAL1 and Ki67 by IHC staining. Data were means ± SD of triplicate determinants (*P < 0.05)

To investigate the biological significance of ST6GAL1 in T-ALL cell lines, the expression of ST6GAL1 was down-regulated in CR/A and JK/A cell lines. As shown in Fig. 3a, ST6GAL1 mRNA and protein levels were decreased in shST6GAL1 cell lines (*P < 0.05). Furthermore, the FITC-SNA lectin on the cell surface was reduced (Fig. 3b). Inhibition of ST6GAL1 attenuated the viability of ADR-resistant cells using CCK-8 assay (Fig. 3c). Average size and number of colonies formed by shST6GAL1 were dramatically smaller than untreated groups by colony-forming unit analysis (Fig. 3d). As a key indicator of cell proliferation, Ki67 was also measured by immunofluorescence staining. Ki67 expressed weak fluorescence intensity in the shST6GAL1 group (Fig. 3e). Knockdown of ST6GAL1 attenuated the chemoresistance with different chemotherapeutic agents using CCK8 assay (Fig. 3f). IC_50_ values were decreased in ST6GAL1 decreasing cell lines (Fig. 3g). Cultured with different drugs, shST6GAL1 cell lines showed lower caspase3 and PARP levels and higher levels of cleaved caspase3 and cleaved PARP (Fig. 3h). The increasing ability of apoptosis was detected by FCM in down-regulation of ST6GAL1 (Fig. 3i). Established xenograft model showed decreased level of ST6GAL1 inhibited tumor growth (Fig. 3j). The levels of ST6GAL1 and Ki67 in xenograft tumor were also verified by IHC staining (Fig. 3k). The quantitive measurement of IHC staining is in Additional file 3: Figure S1A.

**Fig. 4:**
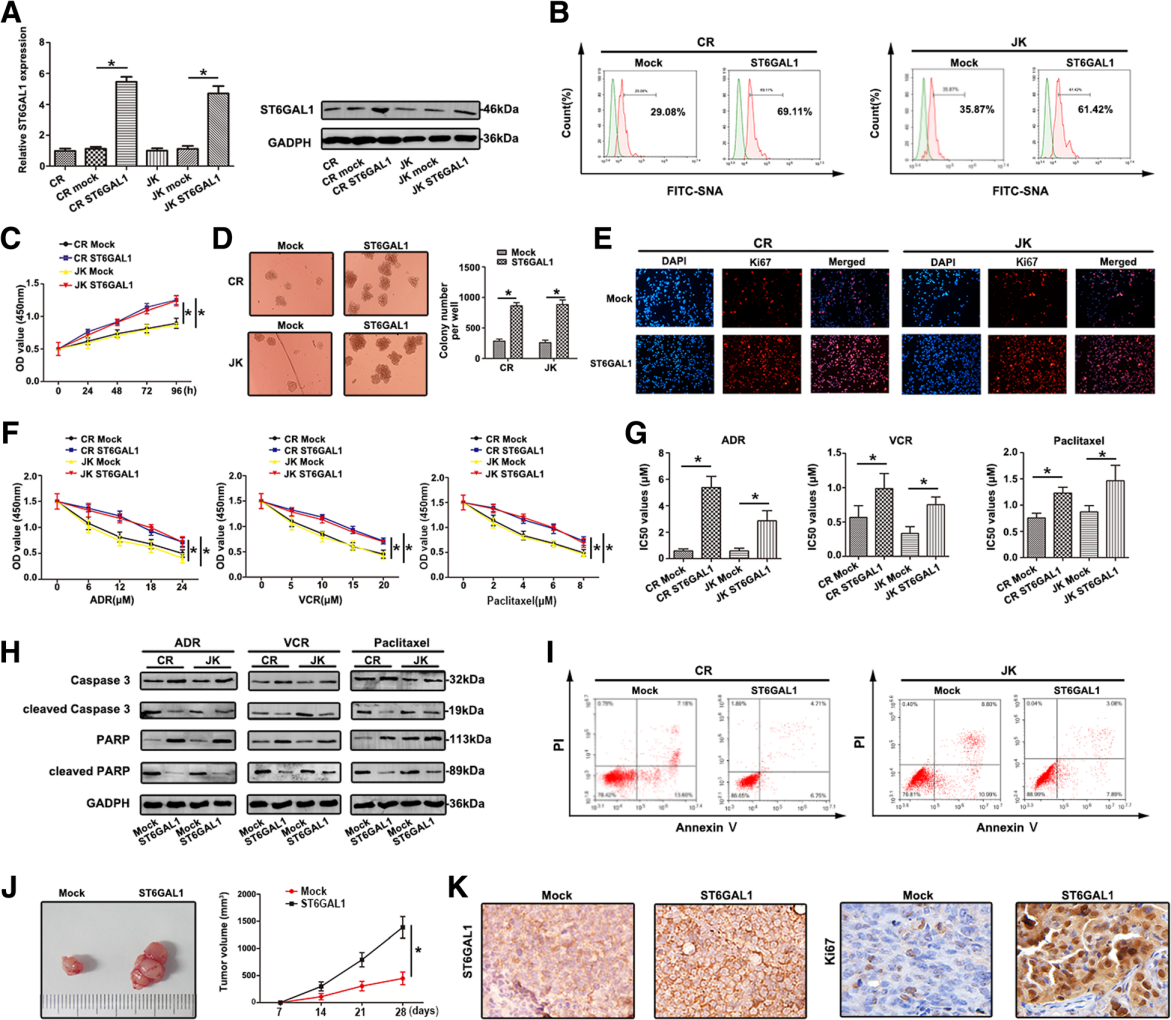
Upregulation of ST6GAL1 promotes proliferation and chemoresistance in T-ALL cell lines (**a**) qRT-PCR and western blot were carried out to detect ST6GAL1 levels. **b** The expression of FITC-SNA was dertermined. **c** The viability of cells transfected with ST6GAL1 was determined by CCK8 assay at 0, 24, 48, 72 and 96 h. **d** Enhanced ST6GAL1 facilitated colony formation. **e** Ki67 expression was observed by immunoflourescence, red fluorescence: Ki67, blue fluorescence: DAPI. **f** CCK8 assays were used to measure the resistance to ADR, VCR and Pacliatxel. The absorbance was measured at 450 nm. **g** The IC_50_ values were calculated. **h** Relative molecular expression of key caspase-dependent apoptosis was analyzed by western blot. **i** The apoptosis rate of different treated cells was analyzed by FCM. ST6GAL1 upregulation inhibited cells survival in response to ADR. **j** Effects of ST6GAL1 upregulation on tumor growth were shown in vivo. **k** ST6GAL1 and Ki67 expression was observed by IHC staining. Data were means ± SD of triplicate determinants (*P < 0.05)

CR and JK cells transfected with ST6GAL1 cDNA caused an increase of ST6GAL1 level compared to parent cells (Fig. 4a). Different expression of α-2, 6 linked salic acids were observed using FITC-SNA lectin. In Fig. 4b, the binding to SNA lectins were higher than mock in transfected CR and JK cells. The up-regulation of ST6GAL1 increased cell viability compared to mock (Fig. 4c). Colony formation assay proved CR and JK cells transfected with ST6GAL1 exhibited higher ability of proliferation (Fig. 4d). Stronger fluorescence intensity of Ki67 was captured after transfecting with ST6GAL1 (Fig. 4e). Furthermore, over-expression of ST6GAL1 promoted cell chemoresistance to multiple chemotherapeutic drugs (Fig. 4f). The IC_50_ values showed similar tendency (Fig. 4g). With different drugs, the levels of caspase3 and PARP were up-regulated, but cleaved caspase3 and cleaved PARP levels showed down-regulation in ST6GAL1 cell lines (Fig. 4h). Decreased apoptosis was shown in overexpressing ST6GAL1 cell lines (Fig. 4i). Nude mice model of ST6GAL1-driven leukemia tumor growth was also assessed. The increase of mean tumor volume was confirmed in CR/ST6GAL1 cells (Fig. 4j). IHC staining was performed to indicate the expression of ST6GAL1 and Ki67 (Fig. 4k). The result of quantitive measurement is in Additional file 3: Figure S1B. These results indicated that ST6GAL1 was responsible for proliferation and chemoresistance through regulating salic acid in T-ALL cells.

### ZFAS1 is a direct target of miR-150 and positively regulates the expression of ST6GAL1 in T-ALL cells

**Fig. 5:**
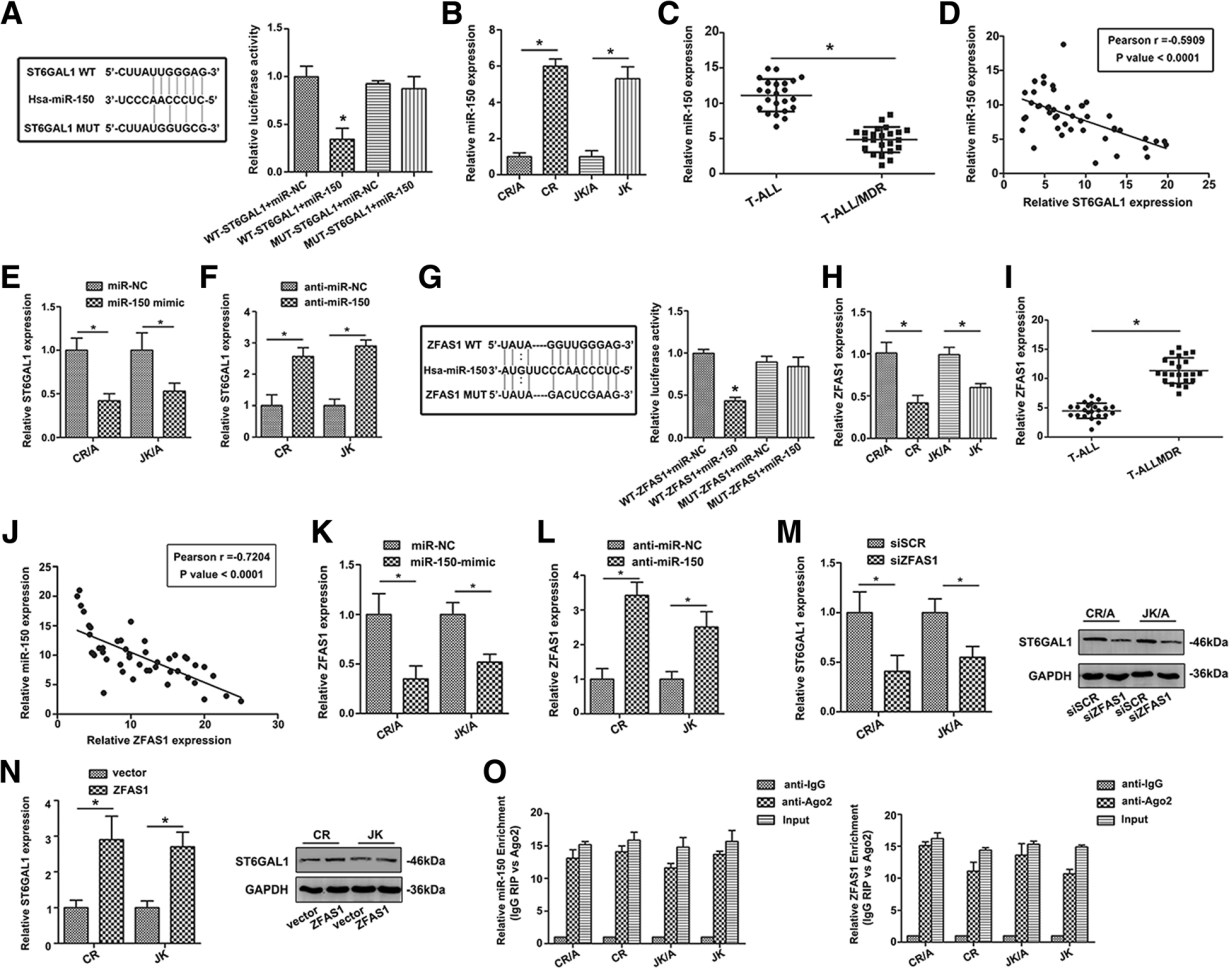
ZFAS1 directly binds miR-150 to regulate ST6GAL1 expression. **a** Sequence aligment of miR-150 with predicted binding sites in the wild-type and mutant-type regions of ST6GAL1 (left panel) was shown. **b** The expression of miR-150 was determined in T-ALL cell lines by qRT-PCR. **c** MiR-150 level was then detected in patients (T-ALL: n = 23, T-ALL/MDR: n = 23). **d** The correlation between ST6GAL1 and miR-150 was determined using Spearman’s correlation analysis. **e, f** The ST6GAL1 expression was shown with miR-150 mimic or inhibitor treatment. **g** Sequence aligment of miR-150 with putative binding sites in sild-type and mutant-type regions of ZFAS1 (left panel) was shown. The wild-type and mutant miRNA target ZFAS1 sequences were fused with luciferace reporter, transfected with miRNA mimic and NC mimic (right panel). **h, i** ZFAS1 expression was determined in T-ALL cell lines and patients by qRT-PCR (T-ALL: n = 23, T-ALL/MDR: n = 23). **j** The Spearman’s correlation was used to analyze the relationship between ZFAS1 and miR-150 expression. **k, l** The ZFAS1 expression was detected by qRT-PCR after cells transfected with miR-150 mimic and inhibitor treatment. **m, n** The ST6GAL1 level was determined in T-ALL cell lines with different treatment of ZFAS1. **o** The co-precipitated RNA was detected by qRT-PCR in RNA immunoprecipitation experiment. ZFAS1 and miR-150 were presented as fold enrichment in Ago2 relative to IgG immunoprecipitate. Data were means ± SD of triplicate determinants (*P < 0.05)

Bioinformatic analysis predicted that miR-150 was closely associated with ST6GAL1. The direct binding sites were confirmed by dual-luciferase reporter gene assay (Fig. 5a). ADR resistant cells and T-ALL/MDR showed decreased level of miR-150 (*P < 0.05, Fig. 5b, c). A significant negative correlation was observed between miR-150 and ST6GAL1 (r = − 0.5909, *P* < 0.0001, Fig. 5d). MiR-150 mimic decreased expression of ST6GAL1 (Fig. 5e), while miR-150 inhibitor could increase ST6GAL1 level (Fig. 5f). ZFAS1 was closely associated with miR-150 through bioinformatics analysis. Luciferase activity in cells co-transfected with mut-ZFAS1 and the miR-150 mimic were comparable to that of control cells (Fig. 5g), confirming that ZFAS1 was a direct target of miR-150. The level of ZFAS1 was measured, and the result showed ZFAS1 level was higher in ADR-resistant cell lines (Fig. 5h). The same change could be observed in T-ALL/MDR group (*P < 0.05, Fig. 5i). Pearson correlation coefficient analysis showed negative correlation between ZFAS1 and miR-150 (r = − 0.7204, *P* < 0.0001, Fig. 5j). ADR-resistant cells transfected with miR-150 showed a lower level of ZFAS1 (Fig. 5k). On the contrary, anti-miR-150 could up-regulate the expression of ZFAS1 (Fig. 5l), indicating that miR-150 was a negative regulator of ZFAS1. In T-ALL cell lines transfected with siZFAS1, a decreasing level of ST6GAL1 was detected (Fig. 5m), while over-expression of ZFAS1 promoted ST6GAL1 level (Fig. 5n).

To further verify whether ZFAS1 was associated with miR-150, RIP assay was performed using anti-Ago2 antibody. ZFAS1 and miR-150 were increased in Ago-2 containing immunoprecipate compared with control immuneglobulin G (IgG) immunoprecipitate (Fig. 5o), providing evidence to association of ZFAS1 and miR-150. GFP-MS2-RIP assay was conducted to confirm miR-150 endogenously associated with ZFAS1 (Additional file 2: Figure S2). Thus, ZFAS1 functioned as a competing endogenous RNA to regulate ST6GAL1 level by sponging miR-150 in T-ALL cell lines.

### ZFAS1 and miR-150 modulate proliferation and chemoresistance through regulating ST6GAL1

**Fig. 6:**
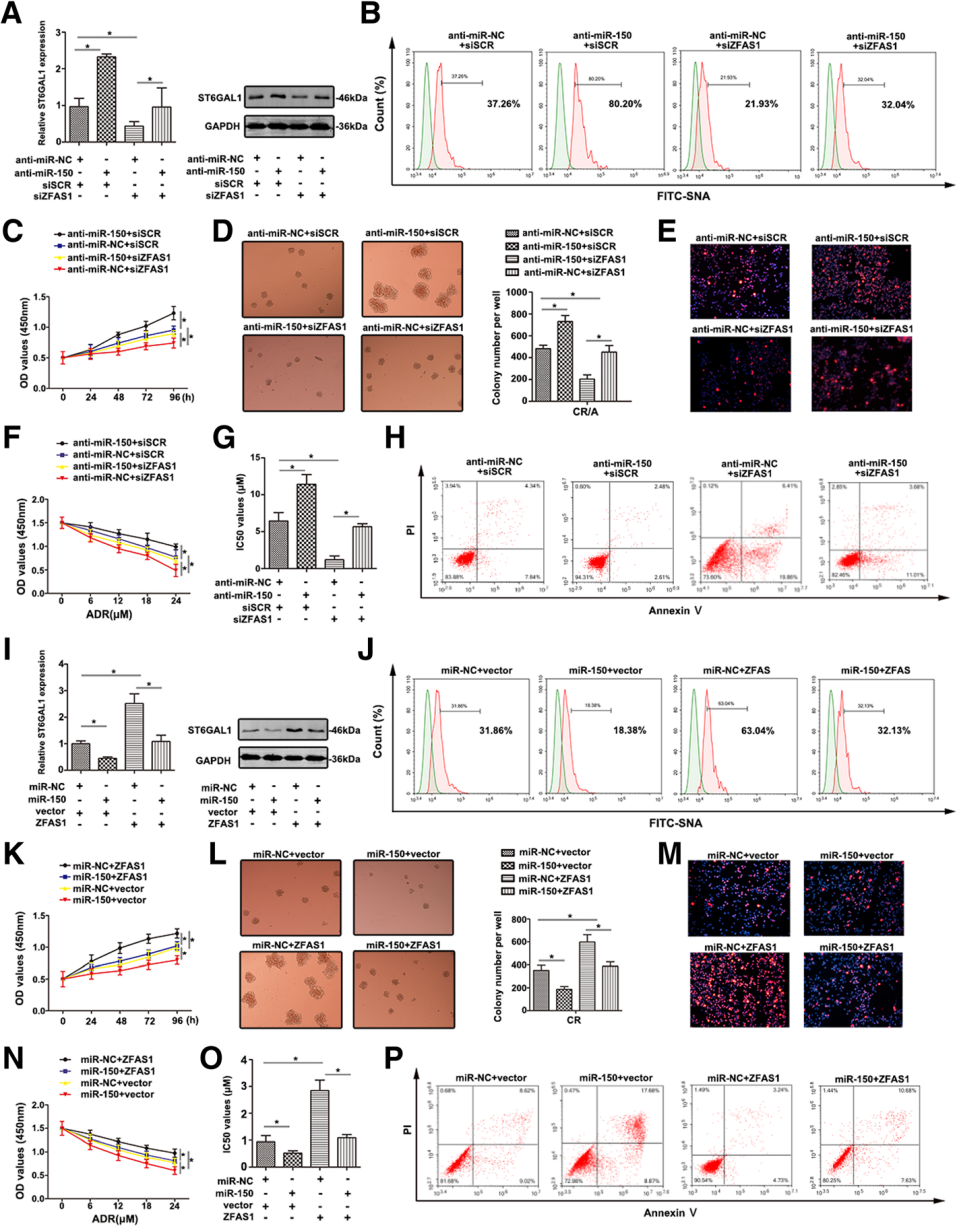
The reversal effect of ZFAS1/miR-150 co-expression on T-ALL chemoresistance (**a**) ST6GAL1 expression was detected by qRT-PCR and western blot with co-transfection siZFAS1 and anti-miR-150 in T-ALL cell lines. **b** The salic acid levels (FITC-SNA) of different treated T-ALL cell lines were shown by FCM. **c**, **d** The proliferative ability of different cells was measured by CCK-8 assay and colony formation analysis. **e** Ki67 expression was showed by immunoflourence staining. **f** The viability of T-ALL cells was detected by CCK8 with ADR treatment. **g** The altered IC_50_ values were calculated. **h** Flow cytometry showed the apoptosis rate of transfected cells in response to ADR. **i** qRT-PCR and western blot were carried out to detect ST6GAL1 expression in co-transfection of ZFAS1 and miR-150 in T-ALL cell lines. **j** FITC-SNA was detected by FCM to show the salid acid level of transfected T-ALL cell surface. **k** The proliferative ability was detected using CCK-8 assay at 0, 24, 48, 72, 96 h. **l, m** Colony formation analysis and Ki67 staining were performed to detect proliferative ability. **n** The cell viability was determined by CCK-8 assay. **o** The IC_50_ values of transfected T-ALL cell lines were calculated. **p** The apoptosis induced by ADR was determined by FCM. Data were means ± SD of triplicate determinants (*P < 0.05)

To clarify the correlation among ZFAS1, miR-150, ST6GAL1 in T-ALL MDR, CR/A cell was transfected with siZFAS1, siSCR, anti-miR-150 and anti-miR-NC. The level of ST6GAL1 was significantly up-regulated by anti-miR-150 and was significantly down-regulated by siZFAS1 (Fig. 6a,*P < 0.05). Anti-miR-150 partially restored the suppression of ST6GAL1 level by siZFAS1 when co-transfection with anti-miR-150 and siZFAS1 (Fig. 6a, lane 4 compared with lane 3, respectively). Similar variation was detected by western blot. In Fig. 6b, anti-miR-150 promoted the α2, 6-sialylation (detected by FITC-SNA lectin), while the sialylated level was down-regulated after ZFAS1 knockdown. More importantly, anti-miR-150 partially reversed the decreased level of sialylation induced by siZFAS1.

The proliferative ability of CR/A cell transfected with anti-miR-150 showed higher than that in other groups, while siZFAS1 could decrease the proliferation (Fig. 6c). Colony-forming assay revealed that anti-miR-150 promoted CR/A cell growth, whereas siZFAS1 inhibited the viability (Fig. 6d). Co-transfection with anti-miR-150 and siZFAS1 showed that anti-miR-150 reversed the proliferation suppressed by siZFAS1. The same tendency was also observed in fluorescence intensity of Ki67 (Fig. 6e). Furthermore, the potential function of ZFAS1/miR-150/ST6GAL1 to ADR resistance was evaluated. Anti-miR-150 CR/A cells became more resistant, while transfected with siZFAS1 remained sensitive. Anti-miR-150 attenuated cell sensitivity to ADR induced by siZFAS1 (Fig. 6f). The similar tendency was proved by calculating IC_50_ values (Fig. 6g). The apoptosis rate showed when co-transfection with anti-miR-150 and siZFAS1, anti-miR-150 restored apoptosis affected by siZFAS1 (Fig. 6h).

ST6GAL1 expression was down-regulated by miR-150 mimic in CR cells, whereas enhanced ST6GAL1 was detected in ZFAS1-overexpressed. MiR-150 abrogated ST6GAL1 level through co-transfection of ZFAS1 and miR-150 (Fig. 6i). Altered sialylation was revealed by fluorescence intensity on FITC-SNA lectin (Fig. 6j). MiR-150 attenuated the viability of CR cells while co-transfection with miR-150 and ZFAS1 strengthened the viability (Fig. 6k). A corresponding effect on colony-formation abilities and Ki67 staining were also observed, further supporting the role of ZFAS1 and miR-150 in mediating ADR-resistant (Fig. 6l, m). With long-term treatment of ADR, miR-150 mimic inhibited CR cell growth, however, up-regulated ZFAS1could reverse the roles (Fig. 6n). The IC_50_ values of ADR on T-ALL cell lines were detected (Fig. 6o). In Fig. 6p, miR-150 increased cell apoptosis while ZFAS1 restored apoptosis partially through co-transfection with miR-150 and ZFAS1. The results elucidated the role of ZFAS1/miR-150/ST6GAL1 axis in the development of T-ALL drug resistance.

### ZFAS1/miR-150/ST6GAL1 axis mediates the sialylation of EGFR and activates the PI3K/Akt pathway

**Fig. 7:**
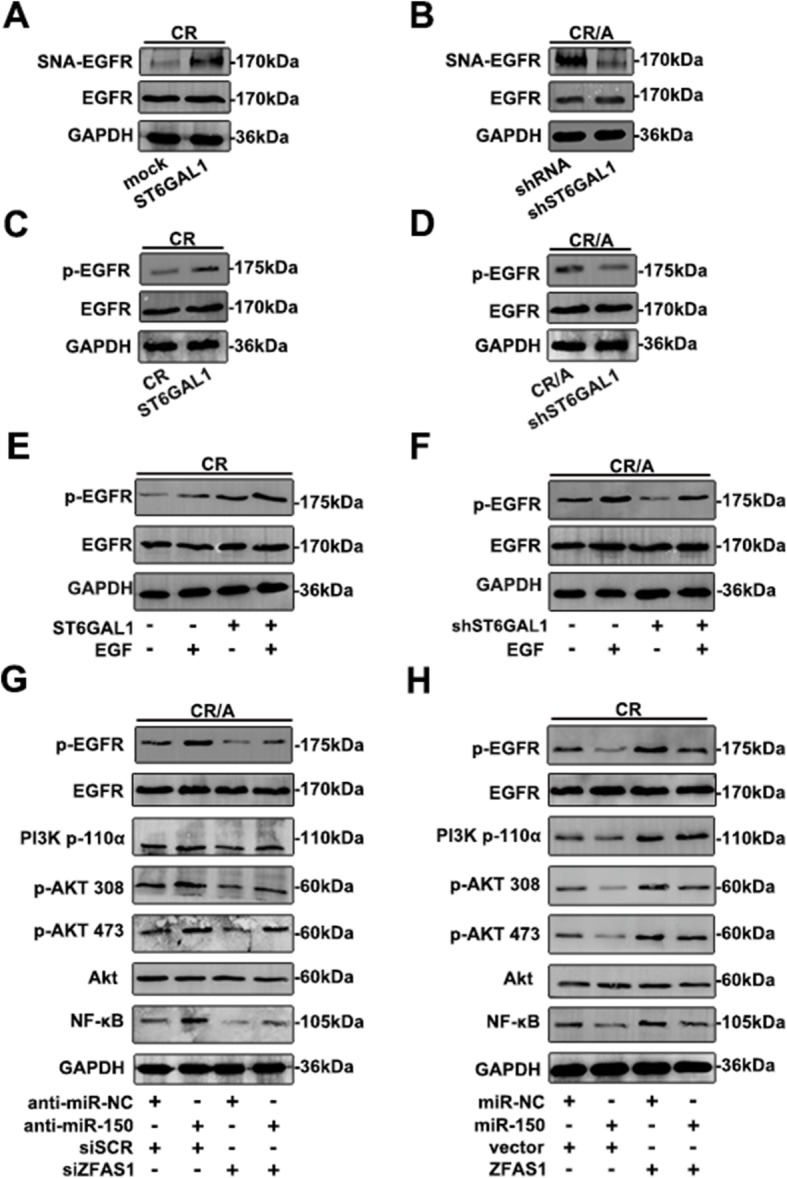
ST6GAL1 facilitates the sialylation on EGFR and activates PI3K/Akt pathway (**a**) CR cell was treated with ST6GAL1. Total EGFR, SNA-binding EGFR were analyzed by western blot. **b** SNA-binding EGFR and total EGFR were analyzed in CR/A cell and transfected shST6GAL1 CR/A cell. **c, d** The expression of p-EGFR and EGFR was measured in T-ALL cell lines treatment with shST6GAL1 or ST6GAL1. **e, f** The p-EGFR level was demonstrated in EGF-treated cells. **g, h** With the mediation of miR-150 and ZFAS1, the main molecular levels of EGFR/PI3K/Akt cascade were detected by western blot

To determine whether EGFR was a direct substrate of ST6GAL1, cell lysates were incubated with agarose-conjugated SNA lectin to precipitate α2, 6-sialylated proteins. As shown in Fig. 7a, α2, 6-sialylated EGFR was upregulated in ST6GAL1 transfected cells. However, the enhanced ST6GAL1 showed no effect on total EGFR level. CR/A cells were transfected with shST6GAL1 to lead to a loss in α2, 6-sialylation on EGFR (Fig. 7b).

Next, to clarify whether the sialylation of EGFR could regulate EGFR signaling, we examined the effect ST6GAL1 on the phosphorylation level of EGFR in T-ALL cell lines. Upregulation of ST6GAL1 facilitated the phosphorylation level of EGFR in CR cells (Fig. 7c), whereas CR/A cells treated with shST6GAL1 showed weaken EGFR phosphorylation (Fig. 7d). In the presence of EGF stimulation, the EGFR phosphorylation was higher in CR cells than the cells without EGF treatment. Surprisingly, CR cells transfected with ST6GAL1 showed stronger phosphorylated degree of EGFR with EGF stimulation (Fig. 7e). However, enhanced p-EGFR was shown in CR/A cells treated with EGF than the control group. In shST6GAL1-CR/A cells, EGF also increased the phosphorylation level of EGFR (Fig. 7f). To further demonstrate whether the EGFR-mediated intracellular pathway was activated in T-ALL cell lines, the key molecular expression was tested. Down-regulated miR-150 promoted the main molecular levels in CR/A cells, including p-EGFR, p-110α, p-Akt308, p-Akt473 and NF-κB (Fig. 7g). Total levels of EGFR and Akt were unchangeable in CR/A cells. Down-regulation of ZFAS1 decreased the phosphorylation of EGFR/PI3K/Akt pathway, while co-transfection with anti-miR-150 reversed the molecular levels, further manifesting a potential regulatory function of ZFAS1 and miR-150. On the contrary, overexpression of miR-150 reduced the activity of EGFR/PI3K/Akt pathway in CR cells, while high expression of ZFAS1 could reverse the situation (Fig. 7h). The results showed ZFAS1/miR-150/ST6GAL1 axis mediated the sialylation of EGFR, and further activated PI3K/Akt pathway.

## Discussion

MS technology as a novel methodology provides high intensity and more rapid glycan analysis. To identify sialyalted *N*-glycan associated with T-ALL MDR, we used MS method to analyze the composition profiling of *N*-glycans. With comparing the total *N*-glycans from CR and CR/A cell lines, remarkable differences was found in *N*-glycan composition. Peaks 22, 24, 25, 28 corresponded to sialylated oligosaccharides from CR/A cells showed a significant increase compared to CR cell (≥2-fold). Therefore, monitoring the sialylated *N*-glycan would be an important step in the prevention of MDR.

Accumulating evidence suggests aberrant expression of sialylated glycans has been detected in numerous cancers [24]. Altered sialylation is usually driven by the dysregulatd STs, which represents an important group of glycosyltransferases. ST6GAL1 is the only STs so far indentified that is able to transfer sialic acid to Galβ1, 4GlcNAc (*N*-acetyllactosamine), a common structure found in *N*-linked chains of glycoproteins [25]. Here, the expression profiles of 20 STs were detected, and the difference of ST6GAL1 was observed in ADR cell lines and PBMCs of T-ALL/MDR patients. Furthermore, increased level of ST8SIA4 was found, consistent with our previous finding in chronic myelocytic leukemia [26]. The result of abnormal expression of ST6GAL1 was similar with previous report that ST6GAL1 was significantly up-regulated in leukemic blasts of ALL compared to normal lymphocytes [10]. Interestingly, increased level of ST6GAL1 was associated with OS in T-ALL patients, indicating that ST6GAL1 might be a survival factor. In addition, altered ST6GAL1 (responsible for sialyaltion) has been correlated with proliferation and MDR in T-ALL cells, supporting the functional involvement of sialylation in T-ALL chemotherapy process. Thus, sialylation and STs could be potential biomarkers in T-ALL MDR.

MiRNAs and lncRNAs were found to participate in the pathological process of tumor drug resistance [11]. Uncovering the mechanisms underneath would be helpful to have a better understanding of T-ALL MDR. Competing endogenous RNA (ceRNA), serving as molecular sponges for miRNA, prevents the subsequent binding of miRNA to target mRNA. Previously, miR-150 was specifically expressed in mature T cells, which played an important role in the development and differentiation of T lymphocytes [27]. ZFAS1 is a newly identified lncRNA, which might serve as a tumor suppressor [28]. Here, abnormal expression of miR-150 was found in ADR resistant cell lines and MDR patients. The negative correlation was found between miR-150 and ST6GAL1 in T-ALL, indicating miR-150 might be involved in the development of T-ALL. ST6GAL1 was a direct target of miR-150 and could be modulated by miR-150. ZFAS1 was inversely correlated to miR-150, and the binding sequence caused miR-150 regulated the expression of ZFAS1. In addition, both ZFAS1 and miR-150 were associated with ST6GAL1. ZFAS1 and miR-150 were also associated with the immunoprecipitated Ago2 complex, and the Ago2 complex cleaved ZFAS1 in the presence of miR-150 in T-ALL cell lines. Interestingly, we also revealed that altered ZFAS1 and miR-150 were significantly associated with ST6GAL1. Consistent with our findings in T-ALL, ZFAS1 was highly expressed in liver cancer, colorectal cancer and was critical for cell metastasis by regulating miR-150 to abrogate its tumor-suppressive function [29]. Moreover, decreased level of ZFAS1 inhibited the proliferation and chemosensitivity of T-ALL cell lines to ADR, while miR-150 inhibitor restored the impact on fundamental cell activity exerted by ZFAS1 inhibition. Logically, the results provided strong evidence that ZFAS1/miR-150/ST6GAL1 axis might modulate the progression of T-ALL MDR.

Recent study discloses that EGFR/PI3K/Akt pathway plays a key role in T-ALL [30]. Constitutive activation of this cascade is associated with aberrant cell survival, metastasis and drug resistance [22, 31]. EGFR signaling has become an important target in anticancer drug development due to its ability to mediate apoptosis, cell proliferation and migration [32]. Recent data reveal that inhibition of EGFR pathway induces cell death in human melanoma and T-ALL cells [33]. Prolonged exposure of the conventional doxorubicin helps to escape cell death of leukemia cells, which induces drug resistance by over-expressing EGFR [34]. The role of sialylation in regulating EGFR has been previously investigated. Park et al. manipulate ST6GAL1 expression in colon carcinoma cells, and reported that EGFR sialylation prevented geftinib-induced cytotoxicity [35]. Clinically, EGFR-TKI has already been widely used to help the patients who take poor reaction in traditional chemotherapy to prolong their life. In current study, we described a new survival function for ST6GAL1 in modulating the activity of EGFR. The manipulation of ST6GAL1 altered the sialylation of EGFR thus influenced phosphorylated EGFR. EGFR was shown to be a direct substrate for ST6GAL1-mediated sialylation, and in every case, α2, 6-sialyaltion of EGFR increased EGF-induced activation of EGFR/PI3K/Akt. Altered miR-150 and ZFAS1 could effectively influence the expression of EGFR/PI3K/Akt pathway molecules. Taken together, these studies highlight the crosstalk of ZFAS1/miR-150/ST6GAL1 mediated T-ALL development through regulating sialylated EGFR via PI3K/Akt pathway.

In conclusion, we revealed the essential role of ZFAS1/miR-150/ST6GAL1 axis in T-ALL development, and demonstrated that ST6GAL1 expression was required for inducing MDR phenotype by regulating sialylated EGFR via PI3K/Akt pathway. ZFAS1/miR-150/ST6GAL1 could be as diagnostic biomarkers and therapeutic targets for T-ALL MDR.

## Conclusion

The regulatory ZFAS1/miR-150/ST6GAL1 axis mediated sialylated EGFR to influence PI3K/Akt pathway in T-ALL MDR. This modulatory crosstalk might be applied as novel biomarker and therapeutic target in T-ALL/MDR.

## Additional files

Additional file 1: **Table S1.** Clinical pathologic characteristics of the T-ALL patients. (DOCX 18 kb)

Additional file 2: **Figure S2.** ZFAS1 is physically associated with miR-150 **(A)** MS2-RIP followed by miR-150 qPCR to assay miR-150 endogenously associated with ZFAS1. (TIF 718 kb)

Additional file 3: **Figure S1.** The intensity of IHC staining (A, B) Quantitive staining intensity of IHC was analyzed by software. (TIF 1074 kb)

## Abbreviations

ADR: Adriamycin
ceRNA: Competing endogenous RNA
LncRNAs: Long non-coding RNAs
MDR: Multi drug resistance
miRNA: MicroRNAs
OS: Overall survival
RIP RNA: Immunoprecipitation
ST6GAL1: α2, 6-sialyltransferase 1
T-ALL: T cell acute lymphoblastic leukemia
VCR: Vincristine
