# Correction to: The regulatory ZFAS1/miR-150/ST6GAL1 crosstalk modulates sialylation of EGFR via PI3K/Akt pathway in T-cell acute lymphoblastic leukemia

**DOI:** 10.1186/s13046-019-1365-y

**Published:** 2019-08-16

**Authors:** Qianqian Liu, Hongye Ma, Xiuhua Sun, Bing Liu, Yang Xiao, Shimeng Pan, Huimin Zhou, Weijie Dong, Li Jia

**Affiliations:** 1 College of Laboratory Medicine, Dalian Medical University, Dalian 116044, 9 Lushunnan Road Xiduan, Dalian, 116044 Liaoning Province China; 2 Department of Clinical Laboratory, Beijing Hospital of Traditional Chinese Medicine Affiliated to Capital University of Medicine Sciences, Beijing, 100010 China; 3 Department of Medicine Oncology, Second Affiliated Hospital of Dalian Medical University, Dalian, 116027 Liaoning Province China; 4 Department of Microbiology, Dalian Medical University, Dalian, 116044 Liaoning Province China; 5 Department of Biochemistry, Dalian Medical University, Dalian, 116044 Liaoning Province China

**Correction to: J Exp Clin Cancer Res (2019) 38:199**

**https://doi.org/10.1186/s13046-019-1208-x**

In the original publication of this article [1], there is a mistake in Fig. 4e.

The corrected Fig. 4e should be:

**Fig. 1:**
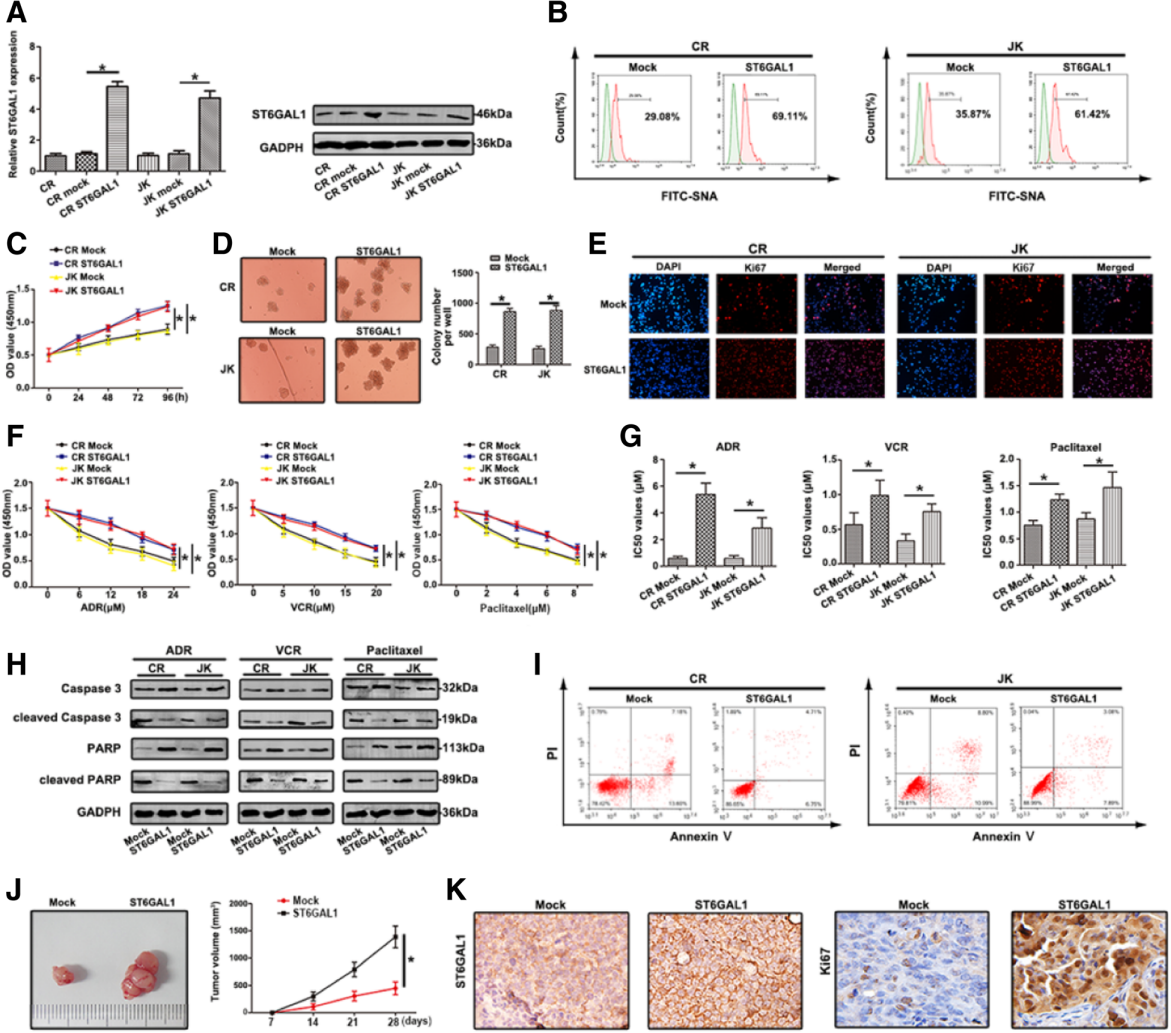
Upregulation of ST6GAL1 promotes proliferation and chemoresistance in T-ALL cell lines (**a**) qRT-PCR and western blot were carried out to detect ST6GAL1 levels. **b** The expression of FITC-SNA was dertermined. **c** The viability of cells transfected with ST6GAL1 was determined by CCK8 assay at 0, 24, 48, 72 and 96 h. **d** Enhanced ST6GAL1 facilitated colony formation. **e** Ki67 expression was observed by immunoflourescence, red fluorescence: Ki67, blue fluorescence: DAPI. **f** CCK8 assays were used to measure the resistance to ADR, VCR and Pacliatxel. The absorbance was measured at 450 nm. **g** The IC50 values were calculated. **h** Relative molecular expression of key caspase-dependent apoptosis was analyzed by western blot. **i** The apoptosis rate of different treated cells was analyzed by FCM. ST6GAL1 upregulation inhibited cells survival in response to ADR. j Effects of ST6GAL1 upregulation on tumor growth were shown in vivo. **k** ST6GAL1 and Ki67 expression was observed by IHC staining. Data were means ± SD of triplicate determinants (*P < 0.05)

## References

[1] Liu Q (2019). The regulatory ZFAS1/miR-150/ST6GAL1 crosstalk modulates sialylation of EGFR via PI3K/Akt pathway in T-cell acute lymphoblastic leukemia. J Exp Clin Cancer Res.

