# Supplementary material for: The regulatory ZFAS1/miR-150/ST6GAL1 crosstalk modulates sialylation of EGFR via PI3K/Akt pathway in T-cell acute lymphoblastic leukemia
Source: J Exp Clin Cancer Res. 2019 May 16;38:199. doi: 10.1186/s13046-019-1208-x (PMC6524305; doi:10.1186/s13046-019-1208-x)
Supplement: Supplementary file 1 — Table S1. Clinical pathologic characteristics of the T-ALL patients. (DOCX 18 kb) [file 13046_2019_1208_MOESM1_ESM.docx]

**Table S1 *Clinical pathologic characteristics of the T-ALL patients***

| Patients demographics | Subcategory | Number (n=46) |
| --- | --- | --- |
| Gender | Male  Female | 30  16 |
| Age (years) | Male  Female | 4-66  6-72 |
| Splenic enlargement | Yes  No | 13  33 |
| Hemoglobin | <110.0g/L  >110.0g/L | 29  17 |
| WBC Count (10^9^/L) | 20-100  >100 | 15  31 |
| Platelet count (10^9^/L) | <100  100-300 | 27  19 |
| P-gp | Positive  Negative | 23  23 |

**Abbreviations: P-gp: P-glycoprotein; WBC: white blood cell;**
