# Supplementary figures and images for: The regulatory ZFAS1/miR-150/ST6GAL1 crosstalk modulates sialylation of EGFR via PI3K/Akt pathway in T-cell acute lymphoblastic leukemia
Source: J Exp Clin Cancer Res. 2019 May 16;38:199. doi: 10.1186/s13046-019-1208-x (PMC6524305; doi:10.1186/s13046-019-1208-x)

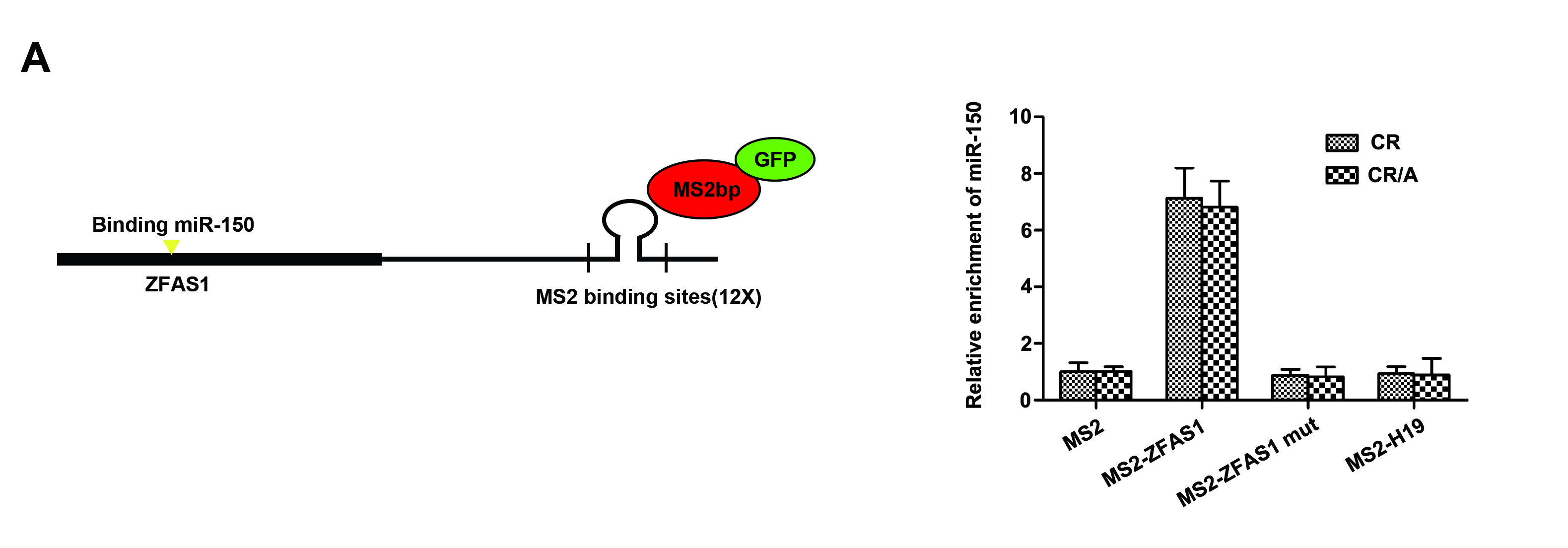

Supplement: Supplementary file 2 — Figure S2. ZFAS1 is physically associated with miR-150 (A) MS2-RIP followed by miR-150 qPCR to assay miR-150 endogenously associated with ZFAS1. (TIF 718 kb) [file 13046_2019_1208_MOESM2_ESM.tif]

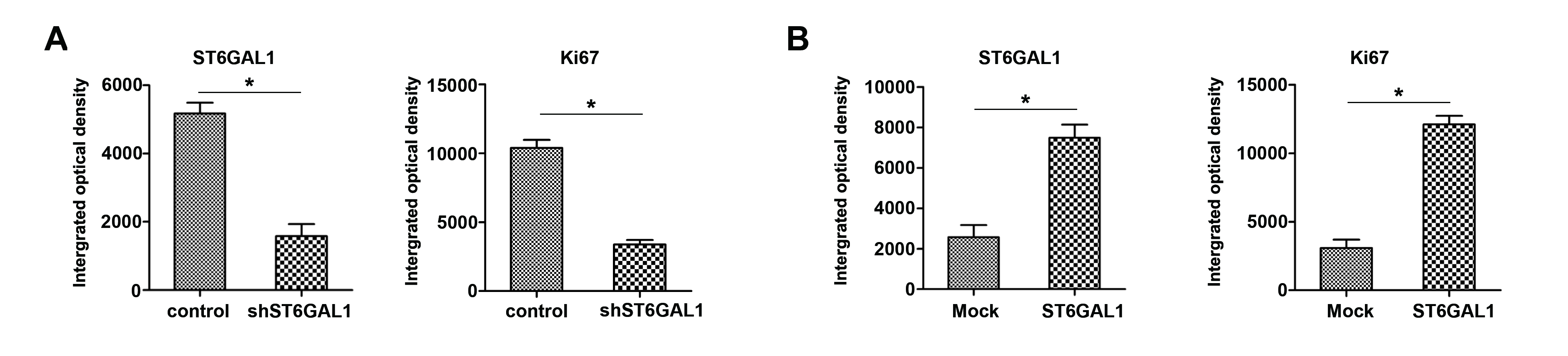

Supplement: Supplementary file 3 — Figure S1. The intensity of IHC staining (A, B) Quantitive staining intensity of IHC was analyzed by software. (TIF 1074 kb) [file 13046_2019_1208_MOESM3_ESM.tif]
